# Thalidomide influences atherogenesis in aortas of ApoE^−/−^/LDLR^−/−^ double knockout mice: a nano-CT study

**DOI:** 10.1007/s10554-014-0380-5

**Published:** 2014-02-01

**Authors:** Marian Kampschulte, Irina Gunkel, Philipp Stieger, Daniel G. Sedding, Anne Brinkmann, Erik L. Ritman, Gabriele A. Krombach, Alexander C. Langheinrich

**Affiliations:** 1 Department of Diagnostic and Interventional Radiology, University Hospital Giessen, Klinikstrasse 33, 35385 Giessen, Germany; 2 Department of Cardiology, Angiology and Pneumology, University Hospital ‘Otto-von-Guericke’ Magdeburg, Magdeburg, Germany; 3 Department of Cardiology and Angiology, Hannover Medical School, Hannover, Germany; 4 Department of Physiology and Biomedical Engineering, Mayo Clinic College of Medicine, Rochester, MN USA; 5 Department of Radiology, BG Clinic Frankfurt/Main, Frankfurt, Germany; 6 Department of Internal Medicine I, Cardiology/Angiology, Giessen University Hospital, Giessen, Germany

**Keywords:** Thalidomide, Atherosclerosis, Angiogenesis, Vasa vasorum, Nano-CT

## Abstract

Plaque progression in atherosclerosis is closely connected to angiogenesis due to vasa vasorum (VV) growth. Objective of this study was to determine the unknown long-term effect of thalidomide on adventitial VV neovascularization and plaque progression using nano-focussed computed tomography (nano-CT). Proliferation and migration assays in human coronary artery endothelial cells (HCAEC) measured number of viable cells after incubation with thalidomide. Male ApoE^−/−^/LDLR^−/−^ (AL) mice (n = 5) received a thalidomide containing western diet (WD) over 29 weeks. Another five male AL mice (WD without thalidomide) served as control group. Descending aortas were scanned with nano-CT at (1.5 μm)^3^ isotropic voxel size. Number and area of adventitial VV as well as plaque cross sectional area were measured. Results were complemented by histology. Thalidomide inhibited proliferation and migration of HCAEC dose-dependently. VV neovascularization decreased in number per cross section (7.66 ± 0.301 vs. 8.62 ± 0.164, *p* < 0.001) and in cross sectional area (0.0183 ± 0.0011 vs. 0.0238 ± 0.0008 mm^2^, *p* < 0.001). Cross sectional area of plaque decreased significantly when treated with thalidomide (0.57 ± 0.0187 vs. 0.803 ± 0.0148 mm^2^, *p* < 0.001). Nano-CT imaging revealed a reduced plaque growth and VV neovascularization after long-term application of thalidomide. Therefore, nano-CT can be considered as a new method to detect therapeutic effects in experimental models of atherosclerosis.

## Introduction

Progression of atherosclerotic plaques, adventitial vasa vasorum (VV) neovascularization and even inflammation are intrinsically tied processes. VV proliferation contributes to the progression of atherosclerotic plaque by maintaining an increased nutrient supply to the vessel wall [1]. In human coronary arteries, VV rupture with concomitant intraplaque haemorrhage is associated with plaque destabilisation [2]. Vice versa, ruptured aortic plaques show an increased microvessel density [3]. Imaging of atherosclerotic lesions for the purpose of discrimination of different plaque stages is imperative for risk estimation in patients with atherosclerosis but still remains a challenge for experimental and clinical CT imaging. Nano-CT represents a technical advancement of micro-CT by using nano focused X-ray tubes. The technique has proven its capability to identify and characterize vascular pathologies with different plaque types, demonstrated VV neovascularisation and plaque growth in aortas of ApoE^−/−^/LDLR^−/−^ double knockout mice (AL mice) [4]. Despite of plaque growth in the ascending aorta and the aortic arch during the lifespan of AL mice, VV density is known to decrease at these sites due to regressive fibro calcification of plaque. In contrast, plaque growth and VV density increases in the descending aorta during the lifespan of AL mice showing instable plaque with intraplaque hemorrhage [4]. Nevertheless, there is no experience in the application of this technique for studying pharmaceutical effects concerning the progress or arrest of atherosclerosis.

Thalidomide is an anti-angiogenic drug [5] with a broad spectrum of current clinical applications ranging from therapy of multiple myeloma [6] to erythema nodosum leprosum [7]. In addition to these clinical applications, different studies proved the molecular effects of thalidomide to reduce systemic and local tumor necrosis factor alpha (TNF-α) production and VV neovascularisation in early atherosclerotic lesions with a concomitant decrease of lesion growth [8–10]. Irrespective of molecular mechanisms and their inhibition by thalidomide, sprouting of microvasculature depends on endothelial cell (EC) migration and proliferation. Currently, the effect of thalidomide on human arterial ECs concerning migration and proliferation is not fully known.

The current nano-CT study was designed to tests the hypothesis that thalidomide (1) influences human coronary artery endothelial cells (HCAECs) function i.e. migration and proliferation, (2) reduces adventitial VV neovasculatization and (3) reduces plaque formation in the descending aorta of AL mice with advanced atherosclerosis. Thereby this study demonstrates the potential of nano-CT as a new and suitable imaging method for preclinical research on therapeutics in atherosclerosis.

## Materials and methods

### Endothelial cell function

To clarify the effect of thalidomide on EC function, cells were incubated in FCS containing growth medium. Thalidomide was dissolved in dimethyl sulfoxide (DMSO) and added in different concentrations to observe whether there is a dose dependent inhibition of proliferation and migration of HCAEC.

### Proliferation

Human coronary artery EC (HCAEC, Cambrex, Verviers, Belgium) proliferation was determined using a BrdU incorporation assay kit (cell proliferation ELISA, Roche, Mannheim, Germany). Briefly, HCAEC were seeded on 1 % gelatin-coated on 96-well plates at a seeding density of 3 × 10^3^ cells per well in endothelial growth medium (EGM-2, Cambrex) for 24 h, followed by serum starvation and pre-treatment with different concentrations of thalidomide between 50 and 250 μg/ml for 24 h. Afterwards cells were stimulated with EGM-2 for 18 h again in the presence or absence of thalidomide in same concentrations. BrdU labeling solution was applied for another 6 h and BrdU incorporation was detected according to the manufacturer’s instructions. Number of viable cells in growth factor enriched buffer alone was set to a value of 100 %. Number of viable cells resulting from different thalidomide concentrations was compared to the number of cells in DMSO-enriched buffer.

### Migration

Human coronary artery endothelial cells (Cambrex, Verviers, Belgium) were grown in endothelial growth medium (EGM-2, Cambrex) at 37 °C and 5 % CO_2_. ECs from passage 2–4 were used for all experiments. Cells were serum starved in 0.5 % fetal bovine serum for 12 h in the presence or absence of thalidomide in concentrations of 500 or 250 μg/ml respectively. Cell migration was measured using a modified Boyden chamber assay. For this purpose, cells were detached with 0.05 % trypsin/0.53 mmol/l EDTA, washed, and the cell suspension (250 μl, 5 × 10^5^ cells/well) was added to the transwell insert (8-μm pore size, Costar, Cambridge, MA, USA). Then, 750 μl of basal medium containing 50 ng/ml human VEGF-A165 (PeproTech Inc, Rocky Hill, NJ, USA) was added to the lower chamber and cells were incubated for 6 h in the presence or absence of different concentrations of thalidomide (500 or 250 μg/ml).

After incubation, non-migrated cells were removed from the upper surface of the insert with a cotton swab. Following reinsertion of transwell inserts, WST reagent (Roche, Mannheim, Germany) was added to the lower chamber and incubated for further 30 min. After removing the inserts, the converted formazan, which directly correlates to the number of viable cells, was quantified in a microplate reader (Tecan sunrise, Crailsheim, Germany) at 450 nm. Number of viable cells in DMSO-enriched buffer alone were set to a value of 100 % and compered to the number of cells resulting from different thalidomide concentrations.

### Animal experiments

Animal studies were performed according to the guideline for animal protection and approved by the institutional animal care and use committee. Thalidomide was added to a fat and cholesterol enriched western type diet (ssniff TD 88137, ssniff Spezialdiäten GmbH, Soest, Germany) and administered with a mean daily dose of 200 mg/kg/day to male AL mice (n = 5, Charles Rivers Wiga, Sulzbach, Germany) beginning at the age of 6 weeks. Coeval male AL mice (n = 5) with the same western diet (WD) but without thalidomide served as controls. Chocolate taste was added to both diets to conceal potentially disgusting taste. Animals were housed in a specific pathogen free environment with access to food and water ad libitum and a 12/12 h day/night cycle. After 29 weeks of diet, i.e. at age of 35 weeks, the animals were euthanized with a fatal dose of inhaled isoflourane. The left ventricle was cannulated and injected with heparinized saline (10 ml of 0.9 % sodium chloride with 1,000 IU heparin) until the venous effluent from the right atrium was free from blood. A plumbiferous radiopaque polymer (Microfil^®^ MV-122, Flow Tech, Carver, MA, USA) was injected into the left ventricle at a nominal pressure of 100 mmHg until it emerged from the inferior vena cava. After polymerization of the compound, the heart and entire aorta were removed and immersed in 4 % neutral buffered formalin.

### Nano-CT

Descending aortas were scanned along the vertical axis using a nano-CT (SkyScan_2011, Bruker Micro-CT, Kontich, Belgium). The scanning system consists of a nano focus, pumped open type X-ray source with a lanthanium boride cathode. The electron beam is focused by two electromagnetic lenses and directed on a tungsten coated beryllium window with a minimum spot size of 400 nm. Tube current was 180 μA and tube voltage was set to produce a 40 kVp transmission X-ray cone beam. Samples were positioned on a computer controlled rotation stage and scanned over 180° in steps of 0.3° around their vertical axis. Acquisition time of each projection image was 2.4 s. For the purpose of noise reduction projection images were acquired repeatedly with averaging of four frames leading to a scan time up to 2 h. The X-ray detector consists of a 12-bit digital CCD high-resolution (1,280 × 1,024 pixel) camera with fibre optic 3.7:1 coupling to an X-ray scintillator and digital frame-grabber. Tomographic reconstruction, using the multi-angular projection image data was performed using a modified Feldkamp cone beam reconstruction algorithm. The resulting voxel resolution was (1.5 μm)^3^ isotropic voxel size.

### Image analysis

A total of 1,084 cross sectional nano-CT images was analyzed. Measurements were performed using the ANALYZE Software packages (ANALYZE 9.0/10.0, Mayo Clinic, Rochester, MN, USA). Atherosclerotic plaque can be detected by virtue of the intraluminal encroachment by the plaque as well as the different X-ray attenuation properties (i.e. CT gray scale) that differentiate the plaque from the intraluminal contrast agent and the normal surrounding tissues of the arterial wall. After blinding of thalidomide-group versus control group to the examiner, VV and atherosclerotic plaques were manually segmented and measured in every tenth subsequent slice of each data set. Quantified parameters were total number of VV, VV cross sectional area (mm^2^) and plaque area (mm^2^) per slice as described previously [4].

### Histology

Subsequent to nano-CT scanning, aortas were dehydrated and embedded in paraffin. Contiguous serial sections (6 μm slice thickness) within each sample were mounted on a microscope slide and stained with haematoxylin and eosin. Masson trichrome identified collagen and elastic fibers used to demonstrate connective tissue (adventitial border). Sequential nano-CT slices were examined for best side-by-side matching of histology and findings of the nano-CT images.

### Statistical analysis

All data are presented as mean ± SEM. Statistical analysis was performed on JMP 6.0 (SAS, Cary, NC, USA). One-way ANOVA, followed by a Tukey–Kramer post hoc test with correction for multiple comparisons was used to identify the statistical differences among groups. Individual group comparison was performed by an unpaired Student *t* test. A value of *p* < 0.05 was considered significant in all analyses. Box–whisker plots were used to illustrate results of statistical findings of nano-CT image analysis, bar graphs were used to demonstrate EC function.

## Results

### Thalidomide inhibits EC proliferation and migration

**Fig. 1:**
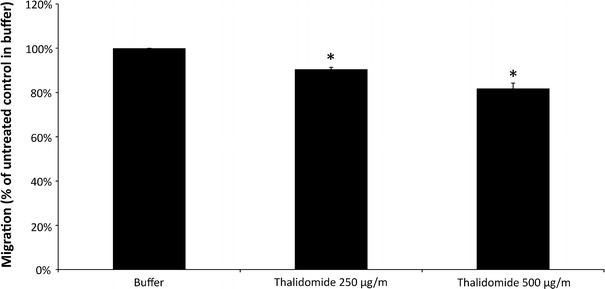
Effect of thalidomide on EC migration: migration of HCAECs is significantly inhibited and dose depending from concentration of thalidomide. *Left column* FCS without thalidomide (control), *middle column* FCS + thalidomide 250 μg/ml; *right column* FCS + thalidomide 500 μg/ml

Compared to growth factor incubated cells, thalidomide significantly inhibited the proliferation of EC dependent to the applicated dose in BrdU assay [42.4 ± 6.6 % (control) vs. 33.4 ± 3 % (thalidomide: 250 μg/m), *p* < 0.05] with findings of significance at concentrations of thalidomide of 100 μg/ml and higher (Table 1). Additionally, migration of ECs was significantly inhibited by thalidomide in a dose dependent manner (*p* < 0.05, Fig. 1) at 250 μg/ml (90.5 ± 0.53 %) and at 500 μg/ml thalidomide (81.8 ± 1.4 %).

**Table 1:** Impact of thalidomide on HCAEC proliferation: BrdU assay of HCAECs in presence or absence of different concentrations of thalidomide

Concentration of thalidomide (μg/ml)	GF	DMSO	Number of cells (%)	Significance
0	+	−	100	–
0	−	+	23.8 ± 5.9	–
0	+	+	42.4 ± 6.6	–
50	+	+	40.3 ± 5.3	n.s.
100	+	+	38.2 ± 4.2	*p* < 0.05
150	+	+	36.9 ± 4.1	*p* < 0.05
200	+	+	34.4 ± 3.2	*p* < 0.05
250	+	+	33.4 ± 3.1	*p* < 0.05

It shows a dose dependent significantly reduced replication of DNA at concentrations of thalidomide at 100 μg/ml and higher, indicating a reduced proliferation

### Thalidomide inhibits VV neovascularization and growth of atherosclerotic lesions

**Fig. 2:**
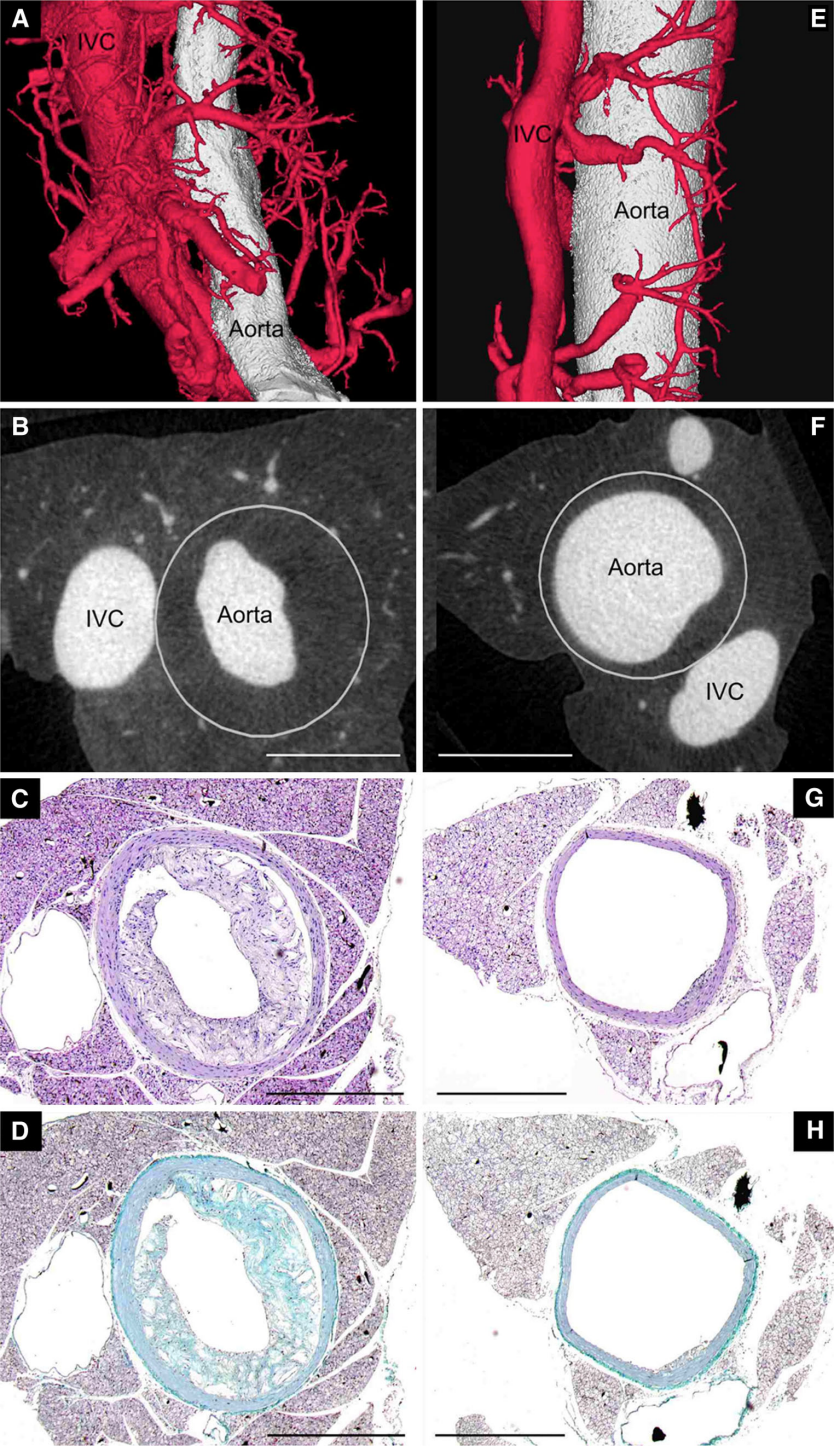
Descending aortas of AL mice at the age of 35 weeks with 29 weeks of administration of thalidomide (*right column*) and control diet (*left column*). 3D volume rendered nano-CT (**a**, **e**) at (1.5 μm)^3^, axial single slices (**b**, **f**), HE-Staining (**c**, **g**) and Masson Trichrome (**d**, **h**) demonstrate the reduced number of VV and the smaller plaque volume in thalidomide treated animals. Corresponding histology depicts the plaque size and adventitial VV locations similar to nano-CT imaging. In contrast to nano-CT, histological preparation incl. cutting leads to artificial disruption of the plaque (**c**, **d**) and tears out polymerized contrast agent (Microfil) from vessel lumen (**c**, **d**, **g**, **h**). The *white circle* defines the border of tunica media to the adventitial tissue

Images obtained by nano-CT enabled a 3D visualization of the aorta and an analysis of plaque characteristics over the entire length of the imaged sample. At the age of 35 weeks, AL mice demonstrate adventitial VV neovascularization and atherosclerotic plaques in the descending aorta (Fig. 2a, b, e, f). This was confirmed by histology (Fig. 2c, d, g, h).

**Fig. 3:**
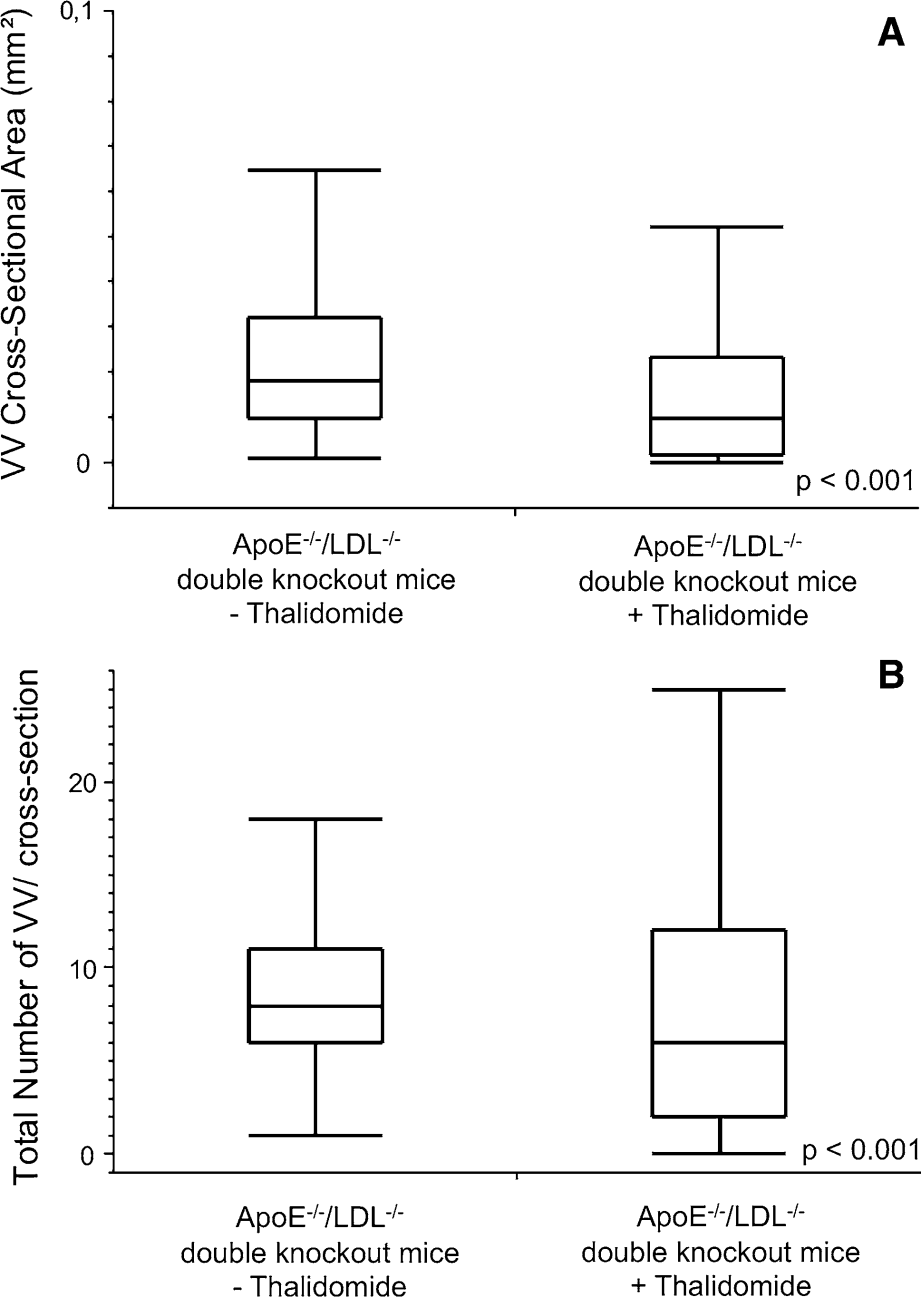
Effects of thalidomide on plaque neovascularization: animals treated with thalidomide demonstrate a significantly reduced total lumen cross sectional area (**a**) and total number (**b**) of adventitial VV compared to controls

**Fig. 4:**
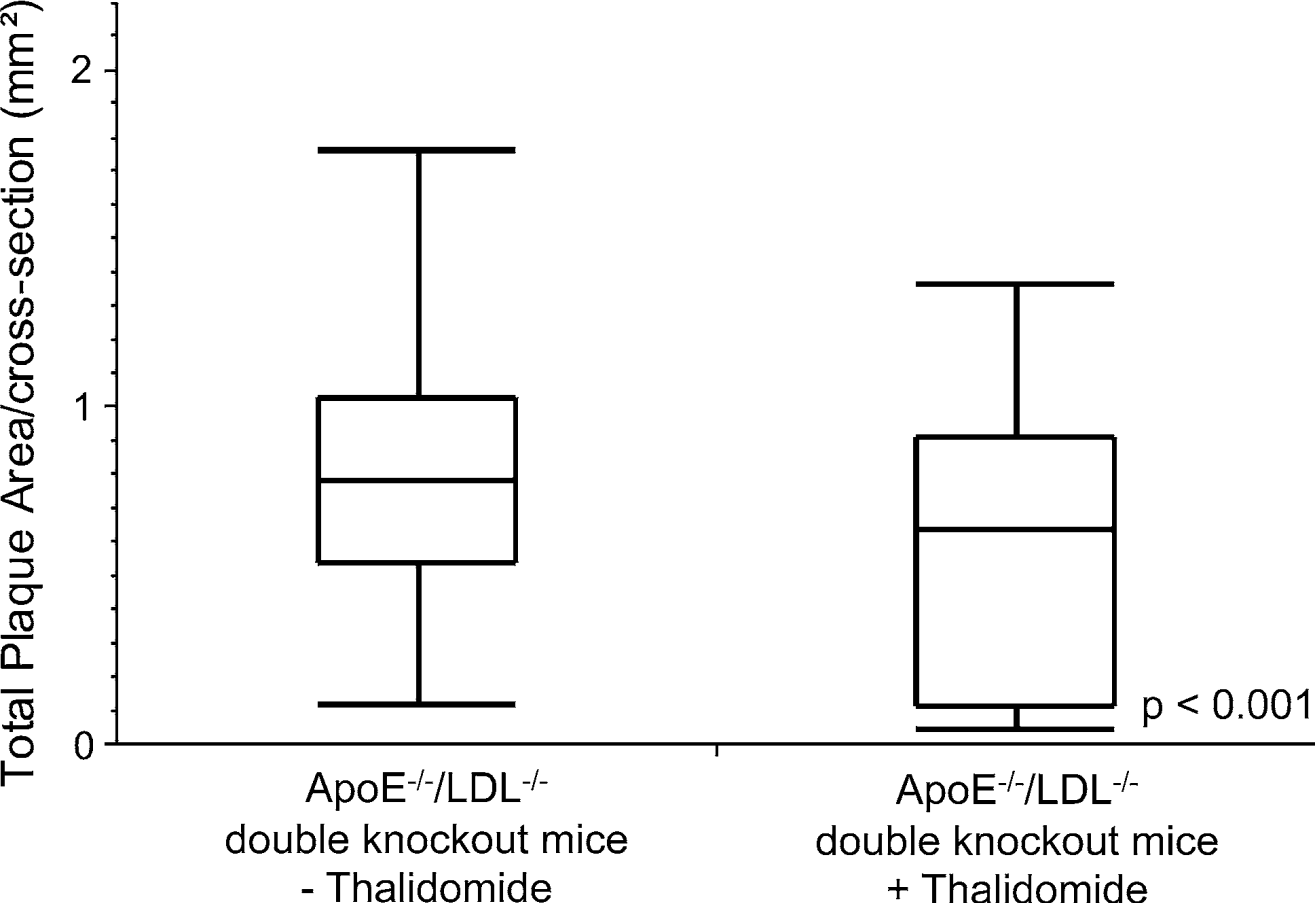
Quantitative nano-CT: effects of thalidomide on plaque size: animals treated with thalidomide demonstrate a significantly reduced cross sectional area of atherosclerotic lesions compared to controls

Compared to controls, quantitative nano-CT data analysis revealed that the cross sectional area of adventitial VV was significantly decreased in thalidomide treated mice (0.0183 ± 0.0011 vs. 0.0238 ± 0.0008 mm^2^, *p* < 0.001, Fig. 3a). This was accompanied by a decrease of the total number of VV per cross sectional image (7.66 ± 0.301 vs. 8.62 ± 0.164, *p* < 0.001; Fig. 2a, b vs. e, f; Fig. 3b). Simultaneously, thalidomide fed animals demonstrated a significant decrease of atherosclerotic plaque area (0.57 ± 0.0187 vs. 0.803 ± 0.0148 mm^2^, *p* < 0.001; Fig. 2b–d vs. f–h, Fig. 4).

## Discussion

The major findings of this study are that thalidomide inhibits (1) proliferation and migration in HCAEC in vitro, (2) adventitial VV growth and (3) growth of atherosclerotic plaque in the descending aorta of AL mice. It supports thalidomide’s effectiveness in an experimental model of atherosclerosis, which shows morphologically comparable findings to human atherosclerosis. The study underlines the value of nano-CT for quantitative imaging of atherosclerosis.

### Thalidomide and endothelial function

Thalidomide was introduced to the European market in 1957 as a sedative and withdrawn in 1961 after press reports about intake of thalidomide during pregnancy and a series of birth defects such as phocomelia or aplasia of extremities. Recent investigations identified cereblon as a protein, responsible for limb outgrowth, which is inhibited by binding to thalidomide [11]. It was banned from therapy over several decades. During the last two decades, it experiences a renaissance due to re-evaluation of its different therapeutic effect. There is evidence that thalidomide influences the expression profile of inflammatory cytokines and angiogenetic factors in different animal and disease models [10, 12–14]. Thereby thalidomide inhibited early neointima formation and reduced VV neovascularization in coronary arteries of WD fed pigs, combined with reduced levels of TNF-α and VEGF [9] and inhibition of early atherogenesis in ApoE deficient mice [8]. Nevertheless, these data do not clarify the impact of thalidomide on function of human ECs neither its influence to plaque and VV growth over a long time span in a model of enhanced atherosclerosis starting at an early time point.

Migration and proliferation of ECs are basic requirements for angiogenesis [15] as seen in VV neovascularization. They can be affected in several ways. In the present study, we demonstrate that thalidomide prevents proliferation and migration of human HCAECs. These findings support previous investigations showing that thalidomide leads to reduced proliferation in human umbilical venous endothelial cells and influences bone marrow angiogenesis in multiple myeloma [16, 17]. It expands the knowledge of thalidomide’s influence on human ECs, showing that it affects arterial as well as venous ECs.

### Quantitative nano-CT for assessment of plaque formation and VV neovascularization

We hypothesized, that a long time application of thalidomide might inhibit lesion progression in concert with VV neovascularization in the descending aorta of AL mice and can be monitored using nano-CT. Similar therapeutic effects were observed for short time application of 3-deazaadenosine to AL mice in early atherosclerosis [18] indicating that growth of atherosclerosic plaque can be (1) influenced in this model of experimental atherosclerosis and (2) monitored using micro-CT. Feeding a thalidomide-enriched WD diet over 29 weeks means a long time exposure of thalidomide to AL mice when assuming a life span of 70–90 weeks in this animal model. At the end of this long time exposure an effect with decreased plaque size was seen. This fact bridges findings concerning the inhibition of early atherogenesis by thalidomide [8, 9] and a late inhibition by angiostatin [19]. Nano-CT proved thalidomide’s influence on VV neovascularization as well as plaque growth in (1) advanced stages of the disease and (2) over a long time span of application. Since plaque type (fibrotic) was the same in both groups, our results indicate a lesser neovascularization i.e. nutrition supply of the vessel wall in concert with thalidomide administration.

For studying this purpose, nano-CT is a versatile and helpful technique that offers additional features for quantitative assessment of atherosclerosis. Just as micro-CT, nano-CT combines non-disruptive ex vivo tissue characterization with 3D visualization and quantification of major vessels (aorta) and its pathognomonic VV micro vascularization. It exceeds micro-CTs spatial resolution to a level of sub micrometer voxel size and provides the opportunity to assess vessels of capillary size. Thereby nano-CT depicts the 3D architecture of the complete vascular tree. In the past, micro-CT measured “density” i.e. changes of gray scale values in atherosclerotic plaque often remained speculative between cellular plaque composition and contrast agent within intraplaque microvessels [20]. Due to its superior spatial resolution, nano-CT can assess pathologies such as intraplaque hemorrhage beyond the limits of micro-CT.

### Limitations

The study was performed in a limited number of animals (n = 5 in each group). Blinded image analysis revealed findings with clear statistical significant differences proving (1) the effect of thalidomide and moreover (2) the applicability of nano-CT for research in atheroscloris. On the other hand, feeding a WD means a metabolic stress leading to obesity and cutaneous xanthoma. Therefore we abstained from a further enlargement of groups. This study proves a reduction of HCAECs viability but was not designed to clarify the specific molecular targets and effects on cellular metabolism in HCAECs. Application of thalidomide began prior to the obvious appearance of VV and atherosclerotic plaque. It needs to be clarified if thalidomide even acts on growth of pre-existing plaque and VV and if it takes influence on the course of the apparent disease simulating a pharmacological intervention after the onset of atherosclerosis. Further research should comparatively investigate molecular similarities and differences of plaque development in AL mice and humans and thalidomide’s mechanistic influence on both. Against this background, 3D imaging with high spatial resolution as provided by nano-CT has the potential to prove the impact of molecular findings on morphological alterations in atherosclerosis.

## Conclusions

Vasa vasorum neovascularization is associated to plaque development. A continued application of thalidomide even over a longer time course reduces both in WD fed AL mice. The data demonstrate nano-CT’s value as a tool to investigate the influence of preclinical drug interventions on atherosclerosis at a microscopic level with 3D data sets in isotropic voxel size. Future research in the field of atherosclerosis inclusive research on novel therapeutics and other topics of microvascular research such as tumor, fracture and inflammation should consider nano-CT as a powerful imaging modality.
